# Comparison of the immersion chilling and freezing and traditional air freezing on the quality of beef during storage

**DOI:** 10.1002/fsn3.2613

**Published:** 2021-10-21

**Authors:** Wanyu Ren, Guoqiang Yuan, Xueer Lin, Xiaohui Guo, Zengli Wang

**Affiliations:** ^1^ College of Food Science and Nutritional Engineering China Agricultural University Beijing China

**Keywords:** beef, frozen storage, immersion chilling and freezing, physical and chemical indexes, traditional air freezing

## Abstract

Compared to traditional air freezing, immersion chilling and freezing shows an improvement in the freezing effect on meat quality, but it is not known whether this advantage persists over longer storage periods. Therefore, the objective of the current study was to compare the effects of immersion chilling and freezing (ICF) and traditional air freezing (TAF) on the physical and chemical indexes in beef longissimus muscle during a storage period of 150 days. In the current study, the longissimus muscle from Luxi cattle (aged 20–24 months) was analyzed, with samples independently frozen by ICF and TAF. After the core temperature was frozen to below −18 degrees by the two chilling methods, samples were transferred to a −18 degrees cold room for further storage. During the storage period, physical and chemical indexes, mainly including color and texture qualities, total volatile base nitrogen (TVB‐N) and peroxide value (POV) were measured and comparatively analyzed at several fixed time points. A higher freezing rate was observed in ICF (5.124 cm/h) than in TAF (0.194 cm/h), and better microstructure was observed in ICF treatment. Besides, peak force values and total energy values were significantly lower in the TAF group than in the ICF group during the first 45 days of freezing storage time (*p* < .05). ICF also showed better color quality due to higher L* values than TAF samples during the first 75 days of frozen storage (*p* < .05). In addition, the thawing loss (after 75 days of storage), total volatile base nitrogen, and peroxide value (in the 30 to 75 days of storage period) were lower in the ICF than in the TAF group. In conclusion, the immersion chilling and freezing is more conducive to the quality of beef during storage at −18 degrees compared to traditional air freezing.

## Graphical abstract

Freezing is one of the most effective ways to preserve the quality of beef and is used to minimize the product loss. Many studies have demonstrated that the immersion chilling and freezing shows good improvement on the freezing effect of meat quality; however, it is not known whether this advantage will still exist in a longer storage period. The aim of current study was to explore the effects of different freezing methods, including the immersion chilling and freezing (ICF) and traditional air freezing (TAF), on the physical and chemical indexes in beef longissimus muscle during a period of 150‐day storage.

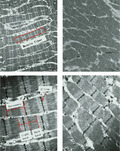

## 1 INTRODUCTION

Frozen preservation is one of the commonly used methods to extend the storage duration of meat products. The freezing processes retard and inhibit biochemical reactions and microbial growth and therefore lead reduction of the deterioration of physical quality and loss of nutritional value in food products during their storage (Cheng et al., 2015). In the past decades, traditional air freezing (TAF), which uses air as the cooling medium to freeze the materials into the −18°C, was a freezing technique widely applied in meat industry. However, the long‐time freezing and high energy waste inhibited the development of TAF. Many studies indicated freezing rate plays a noteworthy role in both of the size and distribution of ice crystals during the freezing processes (Bevilacqua et al., 1979; Bevilacqua & Zaritzky, 1980). Slow freezing rate usually leads to extracellular ice crystals formation in the muscle, which results in damage to muscle proteins and cell membranes (Hergenreder et al., 2013; Martino et al., 1998; Petrović et al., 1993). By contrast, fast freezing may help to the formation of numerous fine ice crystals, which are uniformly distributed in the muscle at a intracellular level, and may decrease the damage to the tissue structure (Mateo‐Oyague & Perez‐Chabela, 2004). Therefore, the freezing rate need to be well controlled to protect the quality of frozen foods (Xiaofei et al., 2016).

The immersion chilling and freezing (ICF) is a fast, instantaneous and energy‐saving freezing technique. ICF uses a multivariate liquid coolant as the heat‐transfer medium, which is characterized by increasing the freezing speed due to the high thermal coefficient of the liquid media (Liang et al., 2015). In addition, ICF could decrease the drip losses, weight loss and maintain the integrity of microstructures. In recent years, many advantages have been found in the frozen processes such as beef, lamb, fish, apples, and broccoli (Delgado et al., 2009; Estrada‐Solís et al., 2016; Fikiin, 1992; Kim et al., 2015; Xin et al., 2014).

Immersion chilling and freezing has showed important impact on the quality of meat products during freezing processing; however, little literature reported how meat quality changes during storage at −18°C condition after different freezing pretreatments. Frozen meat produced by slaughter enterprises is usually sold within 1–3 months. After purchase, many consumers put these frozen meats in the refrigerator at −18°C and then cook them in 1–2 months. Therefore, the aim of current study was to investigate the changes of beef quality under the same storage condition of −18°C in different freezing methods during a period of 150 days.

## 2 MATERIALS AND METHODS

### 2.1 Samples preparation and treatments

Twelve Luxi cattle (aged 20–24 months), originated from the same farm, under the same feeding regime, were slaughtered according to standard routines at a commercial slaughter plant (Hebei Dachang Fuhua Meat Co. LTD). Cattle were weighted between 470 and 510 kg. The carcasses were hung by the Achilles tendon and kept in a chilling room at 4°C for 48 h. The longissimus muscle was cut from the carcasses and used as samples in the current study. Samples were cut into three parts. One part was used for shear force analysis, which was divided into an 18 cm long and caudally distributed from the partitioning site (between the 10th and 11th rib), one part was used for thawing loss, which was divided into pieces (approximately 4 × 1 × 1 cm), and one part was cut into a cuboid about 18 cm long with a 18 ×7 cm cross‐sectional area for other remaining indexes. All of the parts were respectively divided into twenty‐two small parts on average as two kinds of freezing method storage samples. All of the samples were individually vacuum packed and weighed. Samples were randomly located into ICF and TAF groups. The ICF method used special equipment (Beijing Xuteng Xiangyuan Science and Technology Development Co. LTD) to freeze and the temperature of the refrigerant was controlled at −35°C. The TAF group was directly placed in a refrigerator (BD‐266AZF refrigerator Hefei Meiling Co. LTD) at −18°C. For both of the ICF and TAF treatments, samples were transferred into a −18°C refrigerator immediately after the center temperature decreased to −18°C. Physical and chemical detections were performed at a 15‐day interval during a 150‐day storage. And no less than 3 groups of parallel experiments were conducted for each indicator.

### 2.2 Physical indexes

#### 2.2.1 Freezing rate

During the freezing processing, the temperature under both treatments was monitored to draw the freezing curve. Data collection was recorded by a paperless recorder (Shenzhen Jin Shunlong Technology Development Co. LTD), which was fitted with a PT‐100 thermal resistor mounted in sets of two on thin lead wires. One wire was placed into the center of the sample to accurately obtain the reading temperature, and the other wire was inserted under the surface to monitor the surface temperature. The temperature data were recorded at 20‐s intervals. According to the freezing rate definition proposed by the International Institute of Refrigeration in the 1970s, the freezing rate may be represented by the following formula:

$$V_{f}=L/t.$$

where V_f_ is the freezing rate, L is the shortest distance between the surface and the center of the food, and t is the time from 0°C (the food surface temperature) to −10°C (the food core temperature).

#### 2.2.2 Thawing loss

The samples under the two treatments were thawed for 24 h in the air at 4°C after the detection points. Before thawing, the weight of each piece (m_1_) was measured. Subsequently, the pieces were blotted to dry and reweighed (m_2_). The thawing loss was then calculated using the following formula:

$$\text{TL}=\left(m_{1}‐m_{2}\right)/m_{2}\times 100\%.$$

where TL is the thawing loss (%), m_1_ is the frozen weight (g), and m_2_ is the thawed weight (g).

#### 2.2.3 Shear force

The samples were cooked after the respective frozen storage time. The frozen samples were thawed for 24 h in air at 4°C prior to heat treatment. Samples were heat treated in vacuum bags for 2 h in a 70°C water bath in order to make sure to reach the target temperature (70°C) of the samples center, thereafter chill in running cold tap water for 30 min and then store at 4°C until the next day. Shear force was measured using the Warner Bratzler method as described by Honikel (1998).

The beef samples were cut into strips 40 mm long with a 100 mm^2^ (10 ×10 mm) cross‐sectional area. The strips were parallel to the longitudinal orientation of the muscle fibers. And shear force was record as peak force (N) and total energy (mJ). Each sample was measured on a minimum of 12 strips with a CT3‐4500 Texture Analyzer (Brookfield). The texture analyzer was equipped with a Warner Bratzler shear force blade. The cutting blade was 1 mm thick and had the speed of 1 mm/s when cutting through the strips.

#### 2.2.4 Color property

The color parameters were determined using a colorimeter (ADDI, Beijing Chen Tektronix Instrument Technology Co. LTD) with 15 mm aperture, illuminant D65, and 10° observer angle. The color measurements were taken after removing each sample from the vacuum package and exposing it to atmospheric air for 30 min for blooming. Data were recorded using an average of five consecutive measurements representing the entire surface of each sample. The lightness (L*), redness (a*), and yellowness (b*) values were obtained.

### 2.3 Chemical indexes

#### 2.3.1 Total volatile base nitrogen

After removing the fat from the meat samples, 10 g of the meat samples was weighted (0.001 g to be precise), ground with 100 ml water, and then mixed well. The mixed sample was then immersed in a conical flask (for 30 min) and filtered. A 5 ml aliquot of the filtered sample and 5 ml of a magnesium oxide suspension (10 g/L) were distilled using an Automatic Kjeldahl Apparatus KDY‐9820 (Beijing Tongrun Electrical and Mechanical Technology Co. LTD), and the distilled liquid was absorbed with boric acid. Subsequently, the distillate, which included methyl red and methylene blue as indicator, was titrated by standard titrant with hydrochloric acid (0.010 mol/L) until the color of the solution changed from light green to gray or colorless. The experiment was conducted in parallel for 3 times, and the data value of each group (accurate to 0.01 ml) was recorded and the content of volatile basic nitrogen was calculated, and the average value was taken as the Total volatile base nitrogen (TVB‐N) value of beef. The TVB‐N value was calculated according to the following formula:

$$X=\left[\left(V_{1}‐V_{2}\right)\times c\times 14\right]/\left(m\times 5/100\right)\times 100.$$

where X is the TVB‐N value (mg/100 g), V_1_ is the volume of the standard titrant with hydrochloric acid consumed by the sample (ml), V_2_ is the volume of the standard titrant with hydrochloric acid consumed by the blank (ml), c is the actual concentration of the standard titrant with hydrochloric acid (mol/L), and m is the mass of the sample (g).

#### 2.3.2 Peroxide value

The peroxide value of the sample was measured using a modified method of Jung (Jung et al., 2009). The lipids in minced meat samples were extracted from the sample using a mixture of water, methanol, and chloroform (3:5:10). A 1.000 g mass of the extract was dissolved in 25 ml of an acetic acid/chloroform mixture (3:2). Then, saturated potassium iodide (1 ml) was added and the solution was kept in the dark for 10 min. After stabilization, 1.00 ml of starch solution (1 g/100 ml) and 30 ml of distilled water were added to the solution and titrated with 0.01 mol equiv/L Na_2_S_2_O_3_ until the solution became colorless. The experiment was conducted in parallel for 3 times, and the average value was taken as the peroxide value (POV) value of beef. Finally, the POV was calculated using the following formula:

POV(meq/kg)=[(S‐B)×F×molequiv/L(N)]/W×1000.

where S is the titration amount of the sample, B is the titration amount of the blank, F is the titer of 0.01 mol equiv/L Na_2_S_2_O_3_, mol equiv/L(N) is the normality of Na_2_S_2_O_3_, and W is the sample weight (g).

### 2.4 Microstructure

The beef samples were first cut into a cuboid (5 ×3 ×3 mm) along the muscle fiber direction. The prepared samples were then processed as follows: fix in 2.5% glutaraldehyde for 2 h, then cut the fixed meat sample into 3 ×1 ×1 mm pieces with a blade in the direction of the muscle fiber, wash with 0.1 ml phosphate buffer for three times, fix again with 1% osmium tetroxide for 2 h, wash with 0.1 ml phosphate buffer for three times, gradient dehydrate with 30%, 50%, 70%, 80%, 90%, and 100% acetone (in triplicate), embed with epoxy SPURR, polymerized, slice with a LEICA UC6 ultrathin microtome (Leica Germany LTD), dye with 2% uranyl acetate for 30 min, wash with double distilled water, then dye with 6.18% lead citrate for 25 min, rinse with double distilled water, and finally observe using a JEM‐1230 electron microscope (Guangzhou Office of Japan Electronics Co. LTD). At least three repeated observations were collected.

### 2.5 Statistical analysis

All of the experiments were replicated three times. All data were expressed as the means ± standard deviation. Analysis of variance (ANOVA) followed by Duncan's multiple range test was conducted using the Statistical Package for the Social Sciences (SPSS) software (v26.0, SPSS Inc.) to identify any significant differences among the means at a level of *α* = .05.

## 3 RESULTS AND DISCUSSION

### 3.1 Freezing rate

**FIGURE 1:**
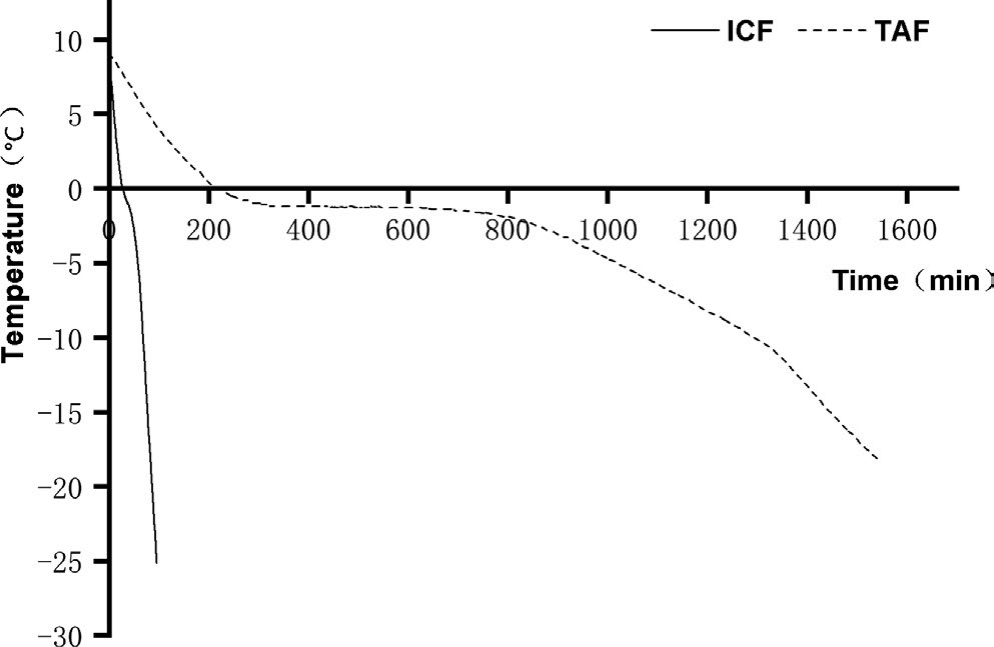
Temperature change curve of the center of beef samples subjected to either immersion chilling and freezing (ICF) or traditional air freezing (TAF) treatments. The solid black line represents the freezing curve of ICF (immersion chilling and freezing) and the dashed black line represents the freezing curve of TAF (traditional air freezing)

Frozen temperature curve under TAF and ICF is shown in Figure 1. The initial center temperature of unfrozen beef was maintained at around 9°C. As the freezing time went on, the center temperatures of ICF samples decreased more sharply than those of TAF samples. Samples of TAF group only decreased to 4.8°C while the center temperature of ICF samples decreased to −18°C at 83 min. The initial characteristic freezing time (Tc) is defined as the time essential for passing from −1.5°C (beginning of freezing) to −7°C, at which 80% of water is frozen in the meat tissue (Bevilacqua et al., 1979). As shown in Table 1, the Tc values were approximately 734 min and 22 min according to TAF and ICF, respectively. In addition, the freezing rate of TAF was 0.194 cm/h, while the freezing rate of ICF was 5.124 cm/h, which was significantly higher than that of TAF.

**TABLE 1:** Freezing rates of beef under ICF (immersion chilling and freezing) and TAF (traditional air freezing)

Freezing method	Freezing time (h)	Distance from surface to heat center (cm)	Freezing rate (cm/h)	Time to pass maximum ice crystal formation zone (min)
TAF	18.017	3.5	0.194	734
ICF	0.683	3.5	5.124	22

The notable difference in freezing rate between the TAF and ICF is closely related to the heat transfer coefficient of the medium. Generally, the thermal conductivity of air is 0.024 W/(m K), while the thermal conductivity of most liquids is 0.116–0.628 W/(m K), which is 5–26 times that of air media. Similar results had been found in other frozen studies such as bighead carp fillets and green beans (Chourot et al., 2001; Qian et al., 2018). The speed of passing through the maximum ice crystal formation zone directly affects the size, number, and distribution of ice crystals in food cells. Specifically, there is negative correlation between the freezing rate and the volume of the ice crystals, and the increment in number of small ice crystals is positively associated with the freezing rate. According to the research of Zhu et al. (2004), when pork was frozen in sucrose‐glycerol refrigerant, the diameter of ice crystals formed in muscle cells was 17 ± 4 μm, while the diameter of ice crystals in pork cells was 93 ± 55 μm when air blast freezing was used, which was much bigger than in liquid freezing. In addition, small ice crystals had been shown to be more effective at preserving the quality of meat products than large ice crystals (Jeremiah & Wilson, 1987).

### 3.2 Thawing loss and shear force

Characteristics of thawing loss between the two freezing methods during storage are shown in Table 2. During the frozen storage, properties of thawing loss were significantly changed in both freezing processes (*p* < .05). And the thawing loss of ICF was significantly lower than that of TAF after 75‐day storage (*p* < .05). The results may be due to that the size of ice crystals in ICF was smaller than in TAF. The thawing loss under both freezing treatments increased with the extension of the storage time. During the frozen storage, the water of the sample would migrate and adhere to the original ice crystals and then form larger ice crystals, which resulted in destroying the ultrastructure of beef. In addition, with the extension of frozen storage time, an increase in thawing loss might result from the increased degree of protein denaturation and the decreased water‐holding capacity of protein (Alonso et al., 2016).

**TABLE 2:** Effect of freezing methods on thawing loss, total energy values, and peak force values during frozen storage for 150 days

Storage time (days)	Thawing loss (%)		Total energy values (mJ)		Peak force values (N)	
	ICF	TAF	ICF	TAF	ICF	TAF
0	2.04 ± 0.50^bA^	2.60 ± 0.20^abA^	109.69 ± 6.73^aA^	91.71 ± 6.31^aB^	42.91 ± 2.99^aA^	37.02 ± 3.46^aB^
15	2.19 ± 0.40^bA^	3.22 ± 0.70^bB^	104.90 ± 12.68^abA^	89.84 ± 11.29^aB^	41.65 ± 2.02^aA^	36.88 ± 2.85^aB^
30	1.80 ± 0.50^bA^	2.19 ± 0.10^aA^	103.54 ± 7.84^abA^	73.00 ± 6.47^bB^	40.07 ± 7.92^aA^	32.87 ± 5.29^abB^
45	0.90 ± 0.24^aA^	3.16 ± 0.30^bB^	99.78 ± 4.46^bA^	68.67 ± 5.59^bcB^	39.82 ± 4.80^aA^	31.86 ± 4.60^abB^
60	4.77 ± 0.60^dA^	5.18 ± 0.50^cA^	85.89 ± 7.50^cA^	57.98 ± 5.59^cdB^	33.29 ± 6.96^bA^	28.58 ± 5.22^bcA^
75	3.74 ± 0.80^cA^	6.11 ± 0.50^dB^	62.37 ± 6.75^dA^	58.45 ± 16.43^cdA^	33.41 ± 3.36^bA^	29.42 ± 7.63^bcA^
90	8.87 ± 0.40^efA^	10.57 ± 0.40^eB^	60.99 ± 5.52^dA^	50.79 ± 21.37^dA^	29.06 ± 3.27^bcA^	25.92 ± 10.93^bcdA^
105	9.49 ± 0.20^fA^	12.50 ± 0.50^fB^	57.45 ± 5.39^dA^	47.20 ± 7.52^dB^	27.13 ± 2.91^bcA^	27.64 ± 5.20^bcA^
120	8.42 ± 0.70^eA^	12.00 ± 0.24^fB^	48.44 ± 8.55^eA^	46.13 ± 8.44^dA^	27.70 ± 9.29^bcA^	25.86 ± 3.24^bcdA^
135	9.52 ± 0.10^fA^	12.50 ± 0.30^fB^	45.98 ± 6.43^eA^	48.00 ± 5.88^dA^	26.18 ± 3.50^cA^	23.88 ± 3.41^cdA^
150	11.30 ± 0.50^gA^	14.94 ± 0.10^gB^	44.87 ± 4.25^eA^	45.27 ± 7.83^dA^	23.70 ± 5.61^cA^	19.43 ± 7.39^dA^

Note

^a‐g^Means with different superscript in a column are significantly different (*p* < .05). ^A,B^Means with different superscript in a row are significantly different (*p* < .05).

Abbreviations: ICF, immersion chilling and freezing; TAF, traditional air freezing.

Changes of total energy values and peak force values during storage are also shown in Table 2. The total energy values and peak force values declined significantly with the extension of storage period. Besides, peak force values and total energy values were also affected by the freezing methods in our study (*p* < .05). For example, TAF sample showed significantly lowered peak force values and total energy values than the ICF sample during the first 45‐day frozen storage time. This demonstrates that the ICF could decrease the tenderness of frozen beef during the initial storage. The result is similar to the research of Hou et al. (2020) whose results indicated that ISF‐treated pork showed lower tenderness than AF. Difference in the shear force may be caused by the length of the frozen storage time and protein denaturation and degradation. This is also attributed to a weakening of the cell membrane as a result of ice crystal formation (Leygonie et al., 2012). This effect not only causes damage to the muscle fibers but also destroys muscle integrity. The impact of freezing rate on the shear force of biological samples has been debated widely, with positive impacts of fast freezing on the shear force being reported. Compared with slow freezing, samples subjected to fast freezing show less resistance to shearing or higher scores of sensory tenderness (Hiner et al., 1945; Petrović et al., 1993).

### 3.3 Color property

The color properties, mainly described by L*, a*, b*, are shown in Table 3. During frozen storage, the treatment and storage time significantly affected L* values (*p* < .05). There was a slight increase in the L* values at the initial storage point, and then the L* decreased with the extension of storage time in both ICF and TAF groups. At the 15‐day storage time, the L* values of TAF samples began to decrease, but the L* values of ICF samples started to reduce at 30‐day storage time. And the L* values of ICF samples were significantly higher than those of TAF samples during the first 75‐day frozen storage time (*p* < .05). Both storage time and treatment (ICF and TAF) affected the a* and b* values on the meat surface. No difference in a* values was found between the two methods in the whole storage periods, while a slight increase was observed in a* values at the beginning of storage. Furthermore, the b* values in TAF samples began to reduce at 15‐day storage time, and b* values in ICF started to reduce at 30‐day storage time.

**TABLE 3:** Effect of freezing methods on meat color stability with lightness (L*), green‐red (a*), blue‐yellow (b*) during frozen storage for 150 days

Storage time (days)	L*		a*		b*	
	ICF	TAF	ICF	TAF	ICF	TAF
0	31.53 ± 0.46^aA^	29.45 ± 0.73^bcB^	17.01 ± 3.61^cA^	19.41 ± 3.01^bcA^	11.96 ± 0.94^cdA^	14.38 ± 1.21^abB^
15	31.86 ± 0.72^aA^	29.64 ± 0.91^bcB^	19.06 ± 0.79^abcA^	22.41 ± 1.90^abcB^	12.84 ± 0.35^bcdA^	15.13 ± 1.01^abB^
30	32.09 ± 4.71^aA^	26.67 ± 0.70^efgB^	22.56 ± 3.81^aA^	22.10 ± 1.93^abcA^	15.56 ± 1.71^aA^	15.11 ± 2.11^abA^
45	30.09 ± 2.05^aA^	25.68 ± 1.37^fghB^	20.25 ± 1.78^abcA^	23.04 ± 3.86^abcA^	13.66 ± 1.37^abcdA^	13.82 ± 1.49^abA^
60	31.36 ± 0.45^aA^	27.95 ± 0.45^cdeB^	22.42 ± 3.12^aA^	19.94 ± 2.16^abcA^	15.12 ± 1.34^abA^	13.04 ± 2.47^bA^
75	31.23 ± 1.19^aA^	27.33 ± 1.65^defB^	19.59 ± 1.22^abcA^	20.80 ± 1.54^abcA^	11.86 ± 0.60^dA^	12.97 ± 1.30^bA^
90	31.09 ± 0.40^aA^	30.22 ± 1.80^bA^	18.48 ± 1.45^bcA^	19.16 ± 2.57^cA^	13.62 ± 2.42^abcdA^	13.80 ± 1.29^abA^
105	27.15 ± 2.00^bA^	24.18 ± 2.13^hB^	22.30 ± 0.88^abA^	22.61 ± 1.22^abcA^	16.10 ± 2.33^aA^	13.11 ± 1.12^bB^
120	26.52 ± 0.75^bA^	25.13 ± 0.60^ghB^	22.45 ± 1.55^aA^	23.27 ± 3.02^abA^	14.64 ± 1.57^abcA^	14.80 ± 2.76^abA^
135	32.10 ± 2.41^aA^	32.70 ± 1.71^aA^	19.96 ± 0.81^abcA^	20.25 ± 1.27^abcA^	14.55 ± 1.56^abcA^	13.38 ± 1.05^bA^
150	29.12 ± 1.40^abA^	29.16 ± 0.68^bcdA^	20.64 ± 3.62^abcA^	23.70 ± 1.55^aA^	13.71 ± 2.34^abcdA^	16.42 ± 1.93^aB^

Note

^a‐h^Means with different superscript in a column are significantly different (*p* < .05). ^A,B^Means with different superscript in a row are significantly different (*p* < .05).

Abbreviations: ICF, immersion chilling and freezing; TAF, traditional air freezing.

The result indicated that ICF beef color change process lagged behind TAF beef. It suggested that ICF could maintain beef color better. The change of meat color is thought to be due to two reasons during frozen storage: one is a reduction in the meat water‐holding capacity, which leads to a decrease in the reflectivity of light on the surface of the meat; the other reason is the chemical changes of meat protein and fat during frozen storage. In this regard, myoglobin plays an essential role in the color stability of meat (Leygonie et al., 2012), and the storage of beef promotes a red‐to‐brown color change caused by the enzyme‐catalyzed oxidation of myoglobin to metmyoglobin (Djenane et al., 2002; Nerín et al., 2006; Sánchez‐Escalante et al., 2003).

In general, freezing process tends to reduce the values of L* (Moore, 1990), especially after a long period of frozen storage (Farouk et al., 2009). But a slight increase in L* values during the initial storage time has been reported in many studies (Lagerstedt et al., 2011; Vitale et al., 2014). According to Lee et al. (2008), the change in the chemical form of deoxymyoglobin (DMb) to oxymyoglobin (OMb) during the blooming of bovine *L. dorsi* muscles resulted in higher L* values. However, some researchers obtained opposite results in their comparison of fresh and thawed beef frozen in an air blast freezer. Both Fernández and Kim reported that longer frozen storage time didn't lead to an increase in L* (Fernández et al., 2007; Kim et al., 2015). The discrepancy between these studies could be attributed to the different freezing time adopted in each study (Leygonie et al., 2012). During initial storage time, a* values of the ICF samples were gradually reducing, probably due to auto‐oxidation of deoxymyoglobin (DMb) to brown metmyoglobin (MMb), induced by the effects of freezing on the protein denaturation and/or lipid oxidation (Aroeira et al., 2017).

### 3.4 Total volatile basic nitrogen and peroxide value

Table 4 shows the effect of the freezing methods on the values of TVB‐N. Both of the ICF and TAF led to a gradual increasement in TVB‐N levels during frozen storage. During the frozen storage, the treatment (TAF and ICF) and storage time significantly affected the TVB‐N (*p* < .05). The values of TVB‐N for the ICF method increased from 6.81 mg/100 g to 15.25 mg/100 g, whereas the TVB‐N for the TAF method increased from 8.80 mg/100 g to 16.54 mg/100 g. Besides, ICF samples had significantly lower TVB‐N values compared to the TAF samples in the whole 120 days of storage period (*p* < .05). The volatile basic nitrogen (TVB‐N) refers to the basic volatile nitrogen compounds such as ammonia, primary amine, secondary amine and so on, which are produced by the degradation of food protein in animal food primarily caused by endogenous proteolytic enzymes and bacterial activities. TVB‐N is a very important index to identify the freshness of meat, and its increase is often accompanied by the decrease of sensory indexes of meat products. The higher the content of such substances, the greater the amount of amino acids decomposed in food, which will not only damage the nutritional value of food, but also make the food have an unpleasant rotten smell in severe cases (Hernandez‐Herrero et al., 1999). These data suggested that ICF had an advantage on TVB‐N compared to TAF.

**TABLE 4:** Effect of freezing methods on total volatile base nitrogen (TVB‐N) and peroxide value (POV) during frozen storage for 150 days

Storage time (days)	TVB‐N (mg/100 g)		POV (meq/kg)	
	ICF	TAF	ICF	TAF
0	6.81 ± 0.60^aA^	8.80 ± 0.69^aB^	1.91 ± 0.05^bA^	1.95 ± 0.32^bA^
15	7.96 ± 0.19^aA^	9.67 ± 0.24^aB^	1.37 ± 0.10^aA^	1.28 ± 0.19^aA^
30	10.00 ± 1.11^bcdA^	12.04 ± 0.57^bcB^	2.49 ± 0.21^cA^	2.87 ± 0.12^cB^
45	9.56 ± 0.85^bcA^	11.49 ± 0.64^bB^	3.03 ± 0.15^deA^	3.57 ± 0.26^dB^
60	9.37 ± 0.43^bA^	12.12 ± 0.59^bcB^	3.39 ± 0.25^eA^	3.88 ± 0.27^dB^
75	9.48 ± 0.34^bcA^	12.49 ± 0.37^bcdB^	3.20 ± 0.07^deA^	3.92 ± 0.15^dB^
90	9.82 ± 0.47^bcA^	13.25 ± 0.46^cdeB^	3.38 ± 0.59^eA^	3.88 ± 0.16^dA^
105	10.76 ± 0.49^cdA^	13.69 ± 1.03^deB^	2.93 ± 0.24^cdeA^	3.87 ± 0.24^dB^
120	11.12 ± 0.96^dA^	14.52 ± 0.56^eB^	3.20 ± 0.15^deA^	4.56 ± 0.10^eB^
135	12.66 ± 1.04^eA^	14.22 ± 0.63^eA^	2.77 ± 0.27^cdA^	4.92 ± 0.83^eB^
150	15.25 ± 0.63^fA^	16.54 ± 1.33^fA^	4.10 ± 0.49^fA^	5.62 ± 0.37^fB^

Note

^a‐f^Means with different superscripts in a column are significantly different (*p* < .05). ^A,B^Means with different superscripts in a row are significantly different (*p* < .05).

Abbreviations: ICF, immersion chilling and freezing; TAF, traditional air freezing.

Table 4 shows the impacts of both freezing methods on POV. According to the results, the POV of ICF group was significantly lower than TAF group in the 30‐ to 75‐day and the last 45‐day storage time. During frozen storage, free water crystallization in the muscle led to an increasing solute concentration, which promoted the increase in the contact area between fat and oxygen in the muscle and speeded up oxidation reaction, and then the POV of the sample increased continuously. The freezing process shows a significant influence on the POV values, which is consistent with the view that oxidation is greater in thawed meat than in fresh meat (Hansen et al., 2004) and that the most appropriate way to freeze meat to minimize oxidation is via fast freezing at low temperature (Dransfield, 1994). The freezing rate has important influence on the muscle structure, the chemical changes of proteins, and the extent of physical cell damage through ice crystal formation, and thus affects oxidation (Leygonie et al., 2012). It is likely that freezing process affects meat oxidation because of damage caused to cellular structures (Wanous et al., 1989), particularly membrane lipids (Monahan et al., 1994). This finding is reflected in a decrease in phospholipids, which allows direct contact between substrates and enzymes (Awad et al., 2006).

### 3.5 Microstructure

**FIGURE 2:**
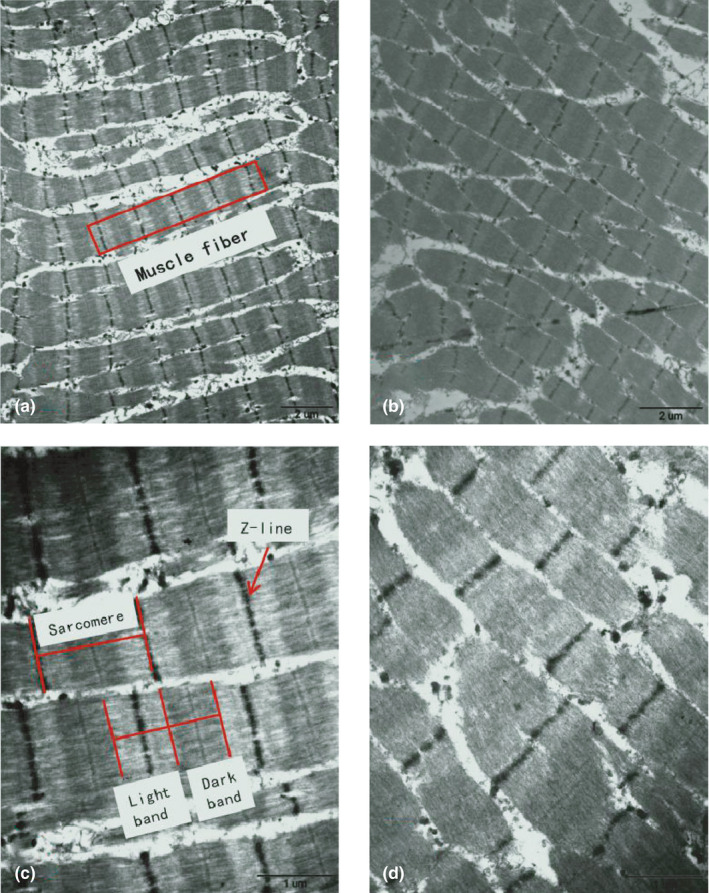
Effect of freezing methods on tissue microstructure. (a) and (c) are images of the muscle fiber structure frozen by immersion chilling and freezing, enlarged by 12,000 and 30,000 times, respectively. (b) and (d) are images of the muscle fiber structure frozen by traditional air freezing enlarged by 12,000 and 30,000 times, respectively

Figure 2a,c show that the myofiber structure was largely intact in the muscle fibers in ICF, with a clear Z line and bright and dark bands. In addition, actin filaments in the bright band and myosin filaments in the dark band could be observed clearly. By contrast, the myofiber structure was collapsed and scraped in the muscle fibers in TAF (Figure 2b,d). Furthermore, the clearance of cells in the image became large, indicating the separation of the muscle fiber and sarcomere, and led to the deformation and shrinkage of the muscle fiber (Figure 2).

During the freezing process, the freezing rate is the primary factor that affects ice crystal formation, and the microstructure of meat would have been affected by this parameter. The effect of ICF is superior to that of TAF because ice crystals formed during air freezing process mainly produce inside the muscle fibers, which generates relatively more compression to the muscle fibers. By contrast, heat transfer in the ICF method occurs rapidly. Consequently, the formation of ice crystals inside and outside the cells occurs concurrently, causing less outward migration of water.

## 4 CONCLUSION

Our results confirmed that beef quality was significantly affected by both TAF and ICF during the storage periods. The freezing rate of ICF was higher than TAF, and the structural properties of beef muscle tissue under the treatment of ICF were better than TAF. Moreover, the shear force and L* values for ICF samples were higher than TAF samples during the first 45 days of frozen storage period. And the thawing loss of ICF was significantly lower than that of TAF after 75 days of storage. Besides, the TVB‐N and POV values of ICF samples were lower than those of TAF samples during the 30 to 75 days of storage period. In conclusion, beef quality during the −18 degrees storage period under ICF treatment was relatively better than TAF.

## CONFLICT OF INTEREST

There is no conflict of interest.

## AUTHOR CONTRIBUTIONS

**Wanyu Ren:** Investigation (equal); Project administration (equal); Supervision (lead); Validation (lead); Visualization (equal); Writing‐original draft (supporting); Writing‐review & editing (lead). **Guoqiang Yuan:** Data curation (equal); Formal analysis (equal); Investigation (equal); Writing‐original draft (equal); Writing‐review & editing (equal). **Xueer Lin:** Conceptualization (lead); Data curation (equal); Formal analysis (equal); Investigation (equal); Methodology (lead); Resources (lead); Writing‐original draft (equal). **Xiaohui Guo:** Project administration (equal); Software (lead); Supervision (equal); Validation (equal); Visualization (equal); Writing‐review & editing (supporting). **Zengli Wang:** Conceptualization (supporting); Funding acquisition (lead); Project administration (lead); Resources (equal); Supervision (supporting); Validation (supporting).

## Data Availability

Data are available on request to the authors.
